# Intermittent normobaric hypoxia at rest is associated with divergent cardiac autonomic trajectories in master cyclists

**DOI:** 10.1007/s00421-026-06280-z

**Published:** 2026-05-29

**Authors:** Pedro Navarro-Gómez

**Affiliations:** Universitat de les Illes Balears, Palma de Mallorca, Balearic Islands Spain

**Keywords:** Heart rate variability, Autonomic modulation, Parasympathetic modulation, Aging athletes, Hypoxic exposure, Crossover design

## Abstract

**Purpose:**

This randomized, participant-blinded, sham-controlled 2 × 2 crossover trial examined whether a 4-week intermittent normobaric hypoxia intervention performed at rest (IHNT; 12 sessions; FiO₂ ≈ 0.12) is associated with changes in indices of cardiac parasympathetic modulation in master cyclists.

**Methods:**

Twenty-three master cyclists aged 50–60 years completed both IHNT and normoxic sham conditions across two periods with a 4-week washout. HRV was assessed from standardized supine morning recordings summarized as 7-day means at Baseline, Post-intervention, and Follow-up. Primary outcomes were lnRMSSD and lnHF, analyzed using linear mixed-effects models with autoregressive residual structure, condition-specific heterogeneous variances, and Holm-adjusted contrasts.

**Results:**

Significant Condition × Time interactions were observed for lnRMSSD (χ²(2) = 16.27, *p* < 0.001) and lnHF (χ²(2) = 18.18, *p* < 0.001), reflecting divergent autonomic trajectories across conditions. Within IHNT, lnRMSSD increased by + 13.6% (*p* < 0.001) and lnHF by + 17.9% (*p* < 0.001) Post-intervention, with partial retention at Follow-up (+ 4.5% and + 7.0%, respectively). Sham showed significant lnHF decreases at Post (− 20.5%) and Follow-up (− 21.3%; both *p* = 0.030), while lnRMSSD remained stable. Pairwise between-condition contrasts did not reach significance after Holm–Bonferroni adjustment. A baseline-adjusted sensitivity analysis confirmed the lnHF interaction (χ²(1) = 12.04, *p* < 0.001). Blinding was maintained (52.4% accuracy, *p* = 0.287).

**Conclusion:**

IHNT at rest was associated with divergent cardiac autonomic trajectories versus sham in master cyclists, with consistent within-IHNT increases in parasympathetic indices and concurrent vagal withdrawal under sham. These proof-of-concept findings support a physiological signal warranting confirmation in adequately powered trials.

**Graphical abstract:**

**Figure:**
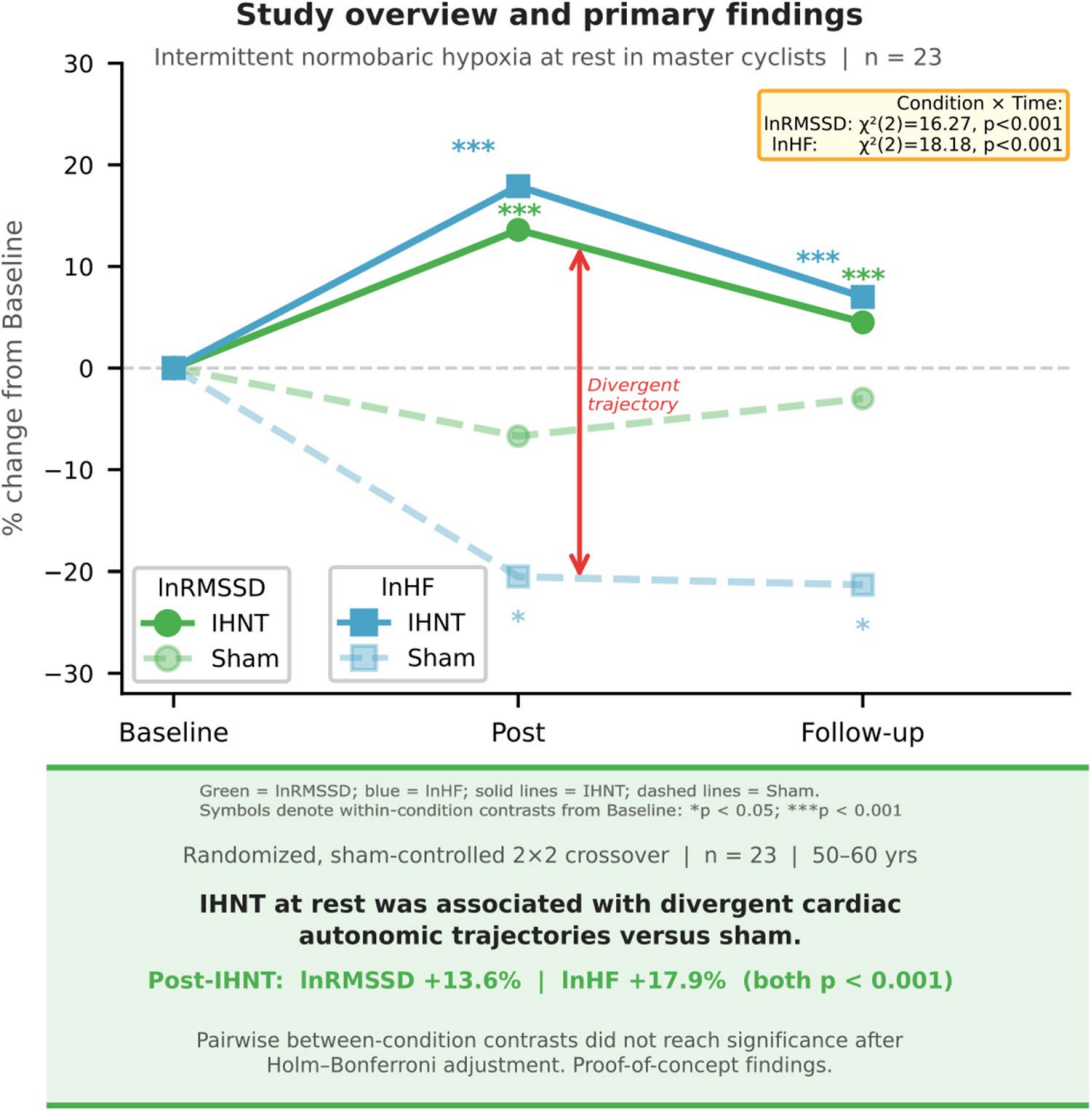

**Supplementary Information:**

The online version contains supplementary material available at 10.1007/s00421-026-06280-z.

## Introduction

Cardiac autonomic regulation represents a fundamental interface between environmental stress, cardiovascular control, and exercise adaptation (Task Force of the European Society of Cardiology and the North American Society of Pacing and Electrophysiology 1996; Shaffer and Ginsberg 2017). In endurance-trained individuals, vagally mediated heart rate variability (HRV) indices—particularly the root mean square of successive differences (RMSSD) and high-frequency (HF) power—serve as noninvasive markers of parasympathetic modulation and recovery status (Task Force of the European Society of Cardiology and the North American Society of Pacing and Electrophysiology 1996; Plews et al. 2013; Shaffer and Ginsberg 2017). Sustained elevations in resting vagally mediated HRV indices are generally associated with favorable training tolerance, whereas sustained reductions may reflect physiological strain (Plews et al. 2013). This autonomic dimension is particularly relevant in master athletes, in whom age-related declines in autonomic responsiveness must be balanced against the demands of long-term training exposure.

Acute hypoxic exposure consistently induces a rapid shift toward sympathetic predominance (Chen et al. 2008; Wille et al. 2012; Krejčí et al. 2018; Ma et al. 2022). Both normobaric and hypobaric hypoxia acutely reduce vagally mediated HRV indices and elevate sympathovagal balance markers (Chen et al. 2008; Wille et al. 2012; Krejčí et al. 2018; Ma et al. 2022). These autonomic alterations exhibit a dose-response relationship with arterial oxygen desaturation (Macoun et al. 2017), with temporal analyses demonstrating that parasympathetic withdrawal occurs within minutes of exposure (Krejčí et al. 2018). Importantly, reduced inspired oxygen fraction (FiO₂), rather than barometric pressure per se, has been identified as the primary determinant of this modulation (Aebi et al. 2020), supporting the translational validity of normobaric hypoxia models.

While acute vagal suppression during hypoxia is well established, the adaptive consequences of repeated, intermittent exposure remain insufficiently characterized (Krejčí et al. 2018; Glazachev et al. 2021; Lee et al. 2025). Emerging data suggest that low-dose normobaric hypoxia may trigger post-exposure rebound increases in vagal indices, consistent with a hormetic autonomic response (Lee et al. 2025). Animal models of chronic intermittent hypoxia demonstrate biphasic autonomic patterns, transitioning from early sympathetic predominance to regulatory normalization (Shi et al. 2020). At the evidence-synthesis level, structured intermittent hypoxia conditioning has been evaluated as a non-pharmacological cardiovascular intervention in clinical and at-risk populations, with autonomic and hemodynamic outcomes among the reported endpoints (Serebrovskaya and Xi 2016; Glazachev et al. 2021). However, this review-level evidence derives from heterogeneous clinical protocols and does not directly establish whether resting IHNT can modify vagally mediated HRV trajectories in trained humans. Collectively, these findings raise a central but unresolved question: can structured intermittent hypoxic stimulation applied at rest induce reproducible parasympathetic reactivation in trained humans?

Human evidence addressing this question is currently fragmented and methodologically constrained. Acute intermittent hypoxia protocols have generally failed to modify resting HRV (Albertus-Cámara et al. 2022; Karayigit et al. 2022), suggesting that isolated exposures are insufficient to alter baseline autonomic tone. Furthermore, many investigations confound hypoxic stimulation with concurrent exercise, making it difficult to isolate the independent effect of hypoxia on autonomic outcomes (Crowcroft et al. 2015; Gronwald et al. 2019) or environmental co-stressors, precluding the isolation of hypoxia-specific autonomic effects. Even recent pilot studies in cyclists have focused primarily on performance outcomes rather than autonomic mechanisms (Peprnicek et al. 2024).

Prior systematic reviews have highlighted substantial methodological heterogeneity in intermittent hypoxia studies, including differences in populations, protocols, outcomes, and analytical approaches (Behrendt et al. 2022; Timon et al. 2023). Parallel-group designs predominate, restricting control over baseline interindividual variability—a major confounder when evaluating adaptive autonomic responses (Botek et al. 2018). Analytically, many prior studies rely on approaches that assume independence of observations and homogeneity of variance, whereas autonomic responses to hypoxic stimuli can exhibit marked interindividual variability (Botek et al. 2018), supporting the use of longitudinal mixed-effects modelling with temporal autocorrelation and condition-specific variance structures (Pinheiro and Bates 2000; Jones and Kenward 2003).

Despite the increasing integration of intermittent hypoxic strategies in athletic settings (Bärtsch et al. 2008), randomized, participant-blinded, sham-controlled crossover trials investigating structured intermittent normobaric hypoxia intervention (IHNT) applied at rest remain extremely scarce in the literature. This gap is particularly evident in master endurance athletes. Therefore, the present study aimed to determine whether a 4-week intermittent normobaric hypoxia intervention performed at rest was associated with divergent vagally mediated HRV trajectories compared with a normoxic sham condition in master cyclists. We hypothesized that IHNT would produce a pattern consistent with greater cardiac parasympathetic modulation than sham.

## Materials and methods

### Ethical approval and participants

The study protocol was approved by the Clinical Research Ethics Committee of the Balearic Islands (CEIm-IB; code IB 5607/24 PI; favorable opinion 27 November 2024; protocol version 1.1, 18 July 2024). All procedures conformed to the Declaration of Helsinki (World Medical Association 2025).

Written informed consent was obtained from all participants prior to participation. The study was pre-registered on the Open Science Framework (OSF) at https://osf.io/2cgxj. The pre-registration specified the study design, hypotheses, primary and secondary HRV outcomes, and the planned mixed-model analytical approach; final model refinements were implemented during blinded analysis.

**Fig. 1:**
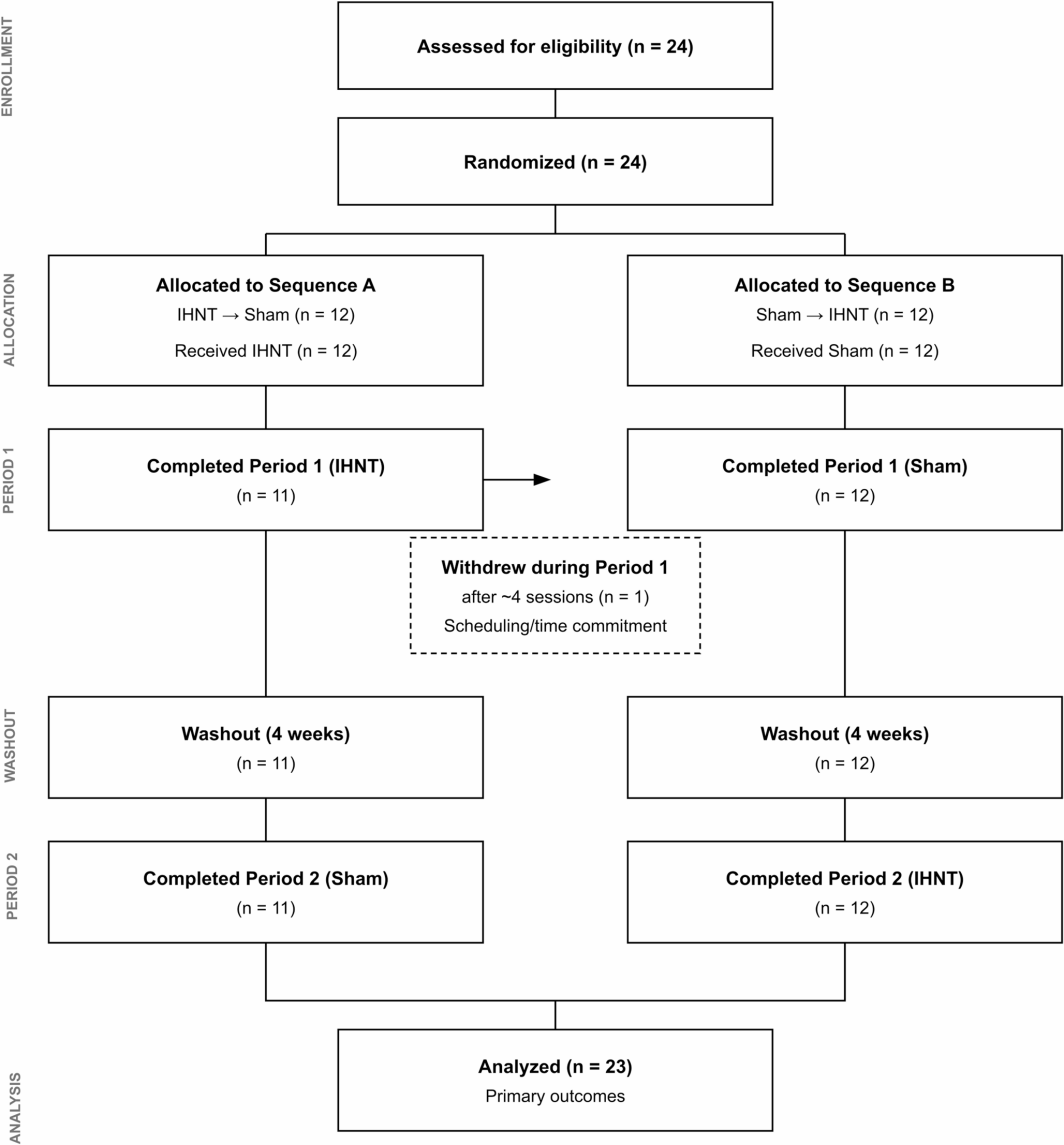
CONSORT flow diagram. Flow of participants through the 2 × 2 randomized, participant-blinded, sham-controlled crossover trial. Twenty-four participants were randomized; one withdrew during Period 1 due to scheduling constraints. Twenty-three participants completed both periods and were included in the primary analyses

Twenty-four master cyclists were recruited and randomized. Inclusion criteria were: age 50–60 years, cycling training volume ≥ 6 h/week for ≥ 12 consecutive months, no diagnosed cardiovascular, pulmonary, or metabolic disease, and resting blood pressure < 140/90 mmHg without antihypertensive medication. This threshold was applied in accordance with European hypertension guidelines applicable in the study context (Williams et al. 2018). Exclusion criteria included: altitude intolerance, sleep apnea, chronic hypoxemia (resting SpO₂ <95%), current smoking or ≥ 10 pack-year history, altitude exposure > 2000 m for > 7 consecutive days within the preceding 6 months, and contraindications to moderate hypoxic exposure. Medical clearance included physical examination, resting 12-lead ECG, and a symptom-limited cardiopulmonary exercise test. Following clearance, participants completed a 10-min normobaric hypoxic tolerance screening (FiO₂ ≈0.12; target SpO₂ ≈80%). One participant withdrew during Period 1 due to scheduling constraints; the remaining 23 completed both conditions and were included in analyses. Participant flow is shown in Fig. 1.

### Study design

**Fig. 2:**
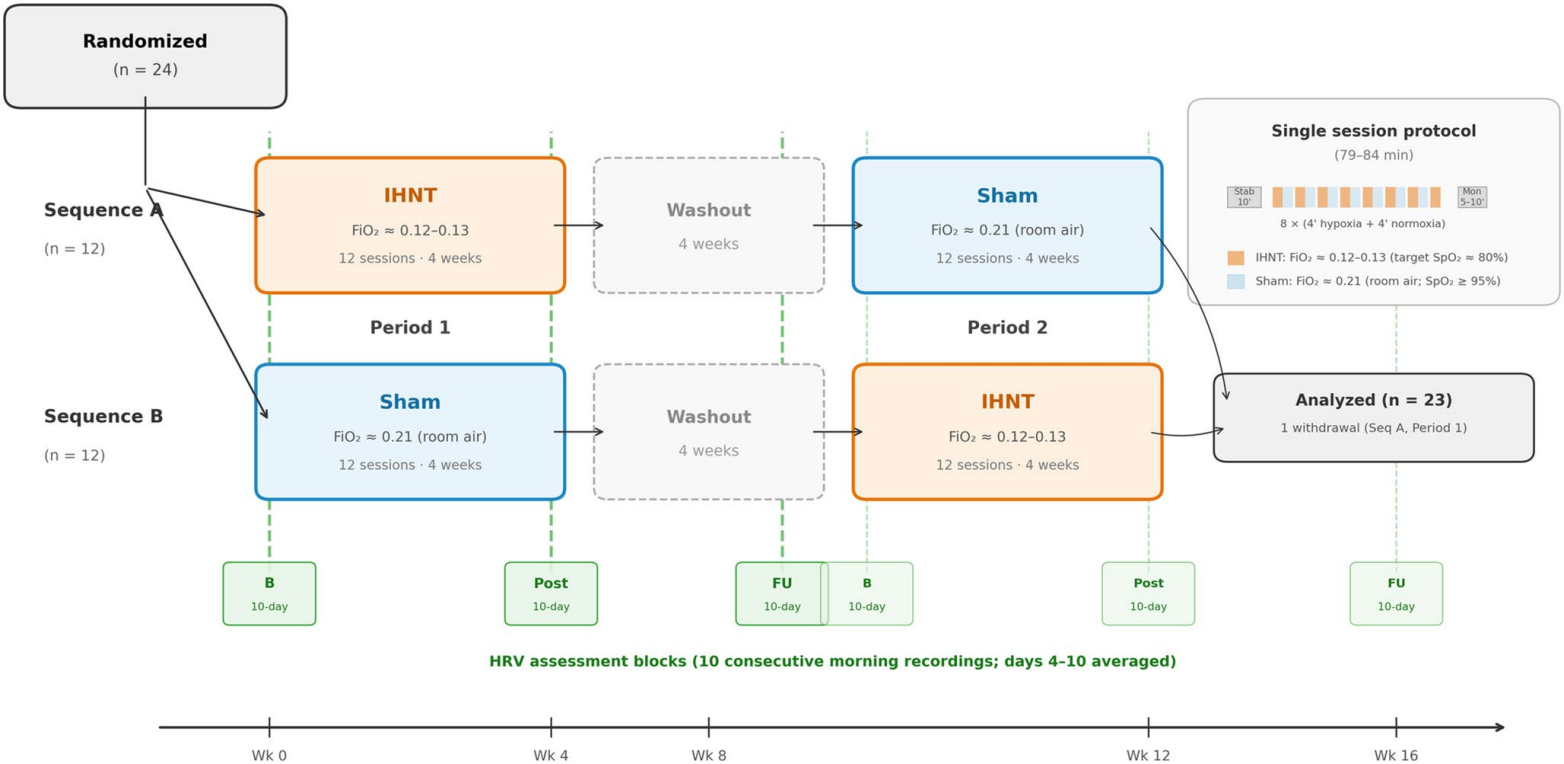
Study design overview for the 2 × 2 crossover trial. Overview of the intervention sequences (IHNT→Sham; Sham→IHNT), 4-week washout, session structure (8 cycles of 4 min exposure interleaved with 4 min normoxic recovery), and HRV assessment blocks (10 consecutive morning recordings; days 4–10 averaged) at Baseline, Post-intervention, and Follow-up within each period

This randomized, sham-controlled, participant-blinded, 2 × 2 crossover proof-of-concept trial compared intermittent normobaric hypoxia intervention performed at rest (IHNT) with a normoxic Sham condition. Participants completed both conditions across two 4-week intervention periods separated by a 4-week washout, serving as their own control. Heart rate variability (HRV) was assessed at Baseline, Post-intervention, and Follow-up within each period (Fig. 2).

Participants were assigned to one of two sequences (IHNT→Sham or Sham→IHNT) using a computer-generated 1:1 randomization schedule prepared by an independent researcher. Allocation codes were concealed from participants, the session operator, and the statistician until database lock. Participants were informed that “oxygen concentrations may vary between sessions” without disclosure of specific FiO₂ values or condition identity. To minimize performance and detection bias, operational roles were segregated among an unblinded technician (generator setup), a blinded session operator (standardized script), and a remote safety monitor (real-time SpO₂ oversight). Blinding success was assessed after each session by asking participants to guess their condition and rate confidence; overall accuracy was 52.4%, not different from chance (*p* = 0.287). Accuracy was calculated across sessions completed by participants who finished both periods (*n* = 23; 24 sessions per participant; 23 × 24 = 552); one participant withdrew during Period 1 after approximately four sessions and was not included in this denominator. Detailed blinding and role-segregation procedures are provided in the Supplementary Material.

### Intervention protocol

Sessions were conducted three times per week on non-consecutive days for four weeks per period (12 sessions per condition) during evening hours (18:00–20:00) in a temperature-controlled laboratory (22 ± 1 °C; 40–60% relative humidity). Participants remained seated in a semi-recumbent position throughout.

Each session comprised: (1) 10-min stabilization (room air); (2) intermittent exposure phase: eight 4-min cycles alternating IHNT or sham gas with 4-min normoxic recovery; and (3) 5–10 min post-exposure monitoring. Total session duration was 79–84 min.

IHNT condition. Normobaric hypoxia was generated using a pressure-swing adsorption system (Longfian JAY-10H, Longfian Scitech Co., China) connected to a 120-L Douglas bag reservoir. Hypoxic gas was delivered via a full-face double-port mask (Hans Rudolph Inc., USA). Delivered FiO₂ was verified with a calibrated portable oxygen analyzer (Maxtec Handi+, Maxtec Inc., USA); mean FiO₂ was 12.3 ± 0.3%. FiO₂ was dynamically titrated to achieve a target SpO₂ ≈80% during each hypoxic interval; the 4-min timer was initiated only when SpO₂ first reached ≤ 82% to standardize effective exposure across participants.

Sham condition. All procedural elements were replicated except inspired oxygen; participants breathed room air (FiO₂ ≈0.21; SpO₂ ≥95%) during exposure intervals.

Safety monitoring. SpO₂ and heart rate were monitored continuously (BCOxygen Oxysleep, Biolaster S.L., Spain; 1 Hz) with Bluetooth transmission to an adjacent monitoring room. Blood pressure was measured immediately before each session, after cycle 4, and immediately after each session. Prespecified stopping criteria included SpO₂ <75% for ≥ 10 s, failure to recover SpO₂ ≥88% during rest intervals, or symptomatic hypoxemia. No sessions met termination criteria and no adverse events occurred.

### HRV assessment

For each assessment phase (Baseline, Post-intervention, Follow-up), participants performed 10 consecutive morning HRV recordings under standardized conditions: supine, immediately upon awakening, after spontaneous voiding, fasted, with spontaneous breathing (Task Force of the European Society of Cardiology and the North American Society of Pacing and Electrophysiology 1996). RR intervals were acquired using a Polar H10 chest strap (Schaffarczyk et al. 2022) transmitted via the Elite HRV application, and processed in Kubios HRV (Tarvainen et al. 2014) with standardized artifact correction.

Primary outcomes were the natural logarithm of the root mean square of successive differences (lnRMSSD) and the natural logarithm of high-frequency power (lnHF; 0.15–0.40 Hz). The standard deviation of normal-to-normal RR intervals (SDNN) was analyzed as an exploratory outcome. Each 5-min recording was obtained following a brief stabilization period.

Data aggregation. To improve reliability and reduce day-to-day noise, each 10-day assessment block was summarized by excluding days 1–3 (acclimation) and averaging days 4–10 (7-day mean), consistent with established HRV monitoring practice (Plews et al. 2013; Buchheit 2014). Assessment timing was: Baseline (10 days preceding intervention), Post-intervention (initiated 48–72 h after the final session), and Follow-up (≈ day 20–21 post-intervention).

### Training load monitoring

Participants maintained habitual cycling training throughout both periods. Weekly cycling volume (km·week⁻¹) was extracted from Garmin Connect or Strava logs and compared descriptively between periods. No systematic differences were observed (Supplementary Table S2).

### Statistical analysis

HRV outcomes were analyzed using linear mixed models (LMM) fitted by restricted maximum likelihood (REML), following the mixed-effects modelling framework described by Pinheiro and Bates (2000). Natural-log transformation was applied to RMSSD and HF power because these HRV indices are strictly positive, typically right-skewed, and often exhibit magnitude-dependent variance. Log transformation was therefore used to improve distributional symmetry, stabilize residual variance, and support multiplicative interpretation of model estimates after back-transformation. SDNN was analyzed untransformed. Each model included fixed effects for Condition (IHNT vs. Sham), Time (Baseline, Post-intervention, Follow-up), their interaction, Period (1 or 2), and a carry-over covariate (Jones and Kenward 2003). A random intercept per participant accounted for between-subject variability.

A first-order autoregressive [AR(1)] covariance structure was applied to residuals across the six chronologically ordered observations per participant. Heterogeneous residual variances were estimated separately for each condition to accommodate observed heteroscedasticity. Degrees of freedom were approximated using the Satterthwaite method (Satterthwaite 1946). Holm–Bonferroni adjustment was applied to prespecified between-condition contrasts (IHNT vs. Sham at each time point) (Holm 1979). Within-condition contrasts are reported with nominal p-values. Given extreme heteroscedasticity between conditions (residual variance ratios up to 164.84), standardized effect sizes were not computed; instead, back-transformed percentage differences [%Δ = exp(Δ) − 1] with 95% confidence intervals are reported as the primary effect measure.

A baseline-adjusted sensitivity model was fitted for lnHF, including the period-specific baseline value as a covariate and restricted to Post-intervention and Follow-up, with a re-indexed AR(1) structure.

Model assumptions were evaluated using Q–Q plots and residuals-versus-fitted plots. Observations with |standardized residual| ≥ 3 were screened and addressed in sensitivity analyses (Supplementary Material). Statistical significance was set at α = 0.05. Analyses were performed in R (version 4.4; nlme and emmeans packages) and cross-validated in SPSS (version 28; MIXED). Data are reported as estimated marginal means (EMM) ± SE with 95% CIs.

### Sample size justification

Sample size (*n* = 23) was determined a priori by feasibility and the proof-of-concept nature of the study. The crossover design reduces between-participant variability; inference was based on model-estimated 95% CIs. No post-hoc power calculations were performed, as observed power is mathematically determined by the p-value and provides no additional information (Hoenig and Heisey 2001).

## Results

### Primary outcome: lnRMSSD

The LMM revealed a significant Condition × Time interaction for lnRMSSD (χ²(2) = 16.27, *p* < 0.001; Table 1), indicating divergent autonomic trajectories between IHNT and Sham. Main effects of Condition (*p* = 0.851) and Time (*p* = 0.462) were not individually significant. Carry-over was non-significant (*p* = 0.431). A period effect was also observed (χ²(1) = 4.258, *p* = 0.039; Table 1).

**Table 1:** lnRMSSD — Type III tests for fixed effects (REML LMM)

Fixed effect	Test statistic	df	*p* value
Condition	χ²=0.035	1	0.851
Time	χ²=1.545	2	0.462
Condition×time	χ²=16.272	2	< 0.001
Period	χ²=4.258	1	0.039
Carry-over	χ²=0.621	1	0.431

lnRMSSD = natural log-transformed RMSSD. Model fitted via REML with an AR(1) repeated covariance structure across the six observations per participant and condition-specific heterogeneous residual variances (Sham/IHNT variance ratio = 84.94)

**Fig. 3:**
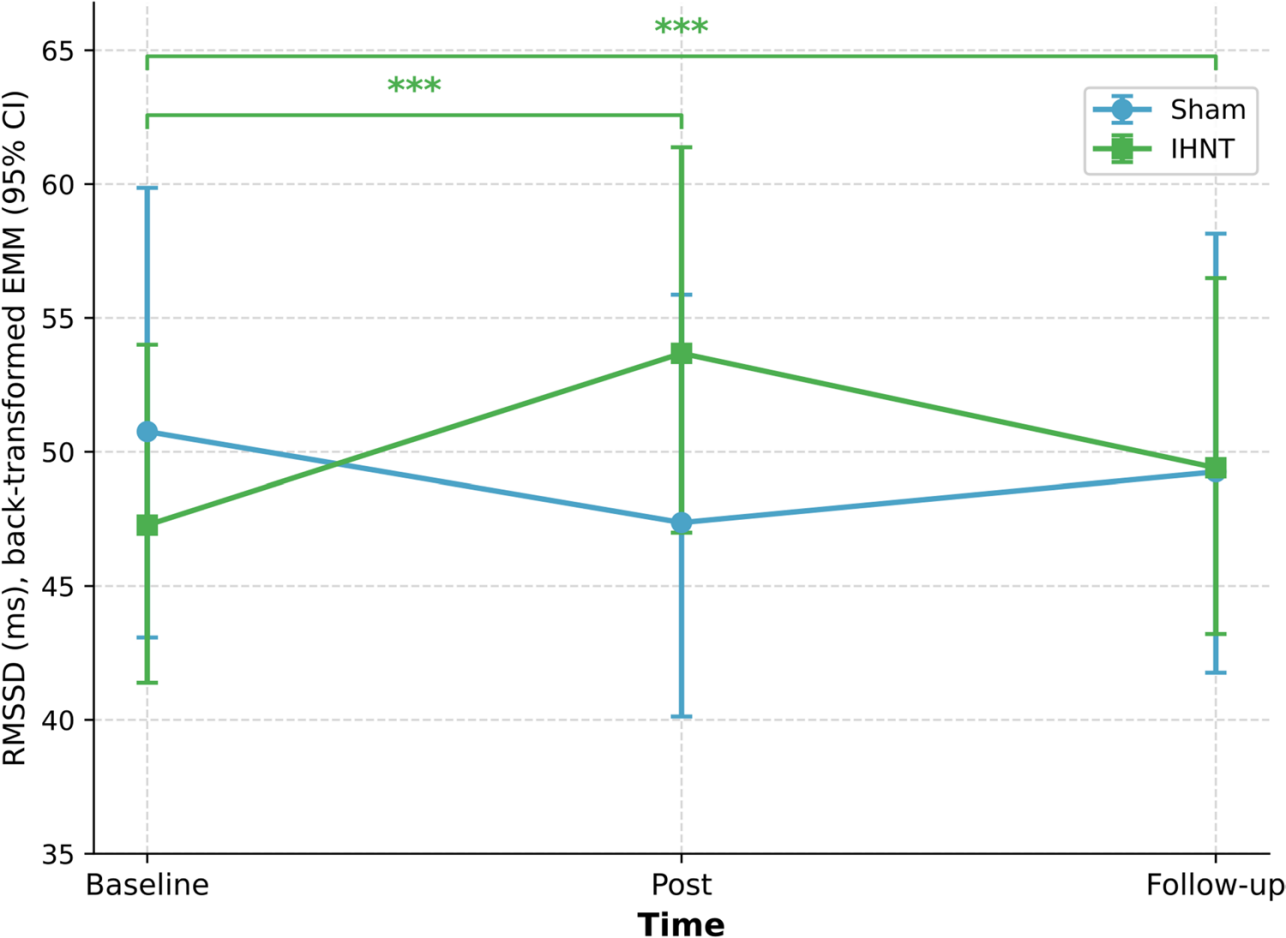
RMSSD profile by condition × time. Model-estimated marginal means (EMMs) with 95% confidence intervals for RMSSD, back-transformed from lnRMSSD, at Baseline, Post-intervention, and Follow-up under IHNT and Sham. Estimates are from the prespecified REML linear mixed-effects model with an AR(1) residual structure and condition-specific heterogeneous residual variances. Symbols denote statistically significant within-condition changes versus Baseline based on nominal p values. No between-condition contrasts reached statistical significance after Holm–Bonferroni adjustment. ***p < 0.001

Within the IHNT condition, lnRMSSD increased from Baseline to Post-intervention by Δ = 0.127 ln units (+ 13.6%; back-transformed 95% CI + 12.1% to + 15.0%; *p* < 0.001) and remained elevated at Follow-up (+ 4.5%; back-transformed 95% CI + 2.9% to + 6.1%; *p* < 0.001). The Sham condition showed no significant within-condition changes at either time point (Post: −6.7%; back-transformed 95% CI − 17.0% to + 4.8%; *p* = 0.358; Follow-up: −3.0%; back-transformed 95% CI − 15.6% to + 11.6%; *p* = 0.626). Back-transformed estimated marginal mean profiles are shown in Fig. 3, with symbols indicating statistically significant within-condition changes versus Baseline.

Between-condition contrasts (IHNT minus Sham) did not reach statistical significance after Holm–Bonferroni adjustment at any time point: Baseline (− 6.9%; back-transformed 95% CI − 24.3% to + 14.6%; *p* = 0.497), Post-intervention (+ 13.4%; back-transformed 95% CI − 8.0% to + 39.7%; *p* = 0.236), and Follow-up (+ 0.3%; back-transformed 95% CI − 18.7% to + 23.6%; *p* = 0.981). Residual diagnostics were consistent with model assumptions; two observations with |standardized residual| ≥ 3 were addressed in sensitivity analyses (Supplementary Table S6).

### Secondary outcome: lnHF

The Condition × Time interaction for lnHF was significant (χ²(2) = 18.18, *p* < 0.001; Table 2). Carry-over was non-significant (*p* = 0.622). A period effect was also observed (χ²(1) = 5.165, *p* = 0.023; Table 2).

**Table 2:** lnHF — Type III tests for fixed effects (REML LMM)

Fixed effect	Test statistic	df	*p* value
Condition	χ²=0.322	1	0.570
Time	χ²=2.755	2	0.252
Condition×time	χ²=18.180	2	< 0.001
Period	χ²=5.165	1	0.023
Carry-over	χ²=0.244	1	0.622

*lnHF* = natural log-transformed HF power (0.15–0.40 Hz). The model was fitted via REML with an AR(1) repeated covariance structure across the six observations per participant and condition-specific heterogeneous residual variances (Sham/IHNT variance ratio = 164.84). Confidence intervals back-transformed to percentage change are asymmetric because of the non-linear exponential transformation; statistical inference was performed on the log-transformed scale

**Fig. 4:**
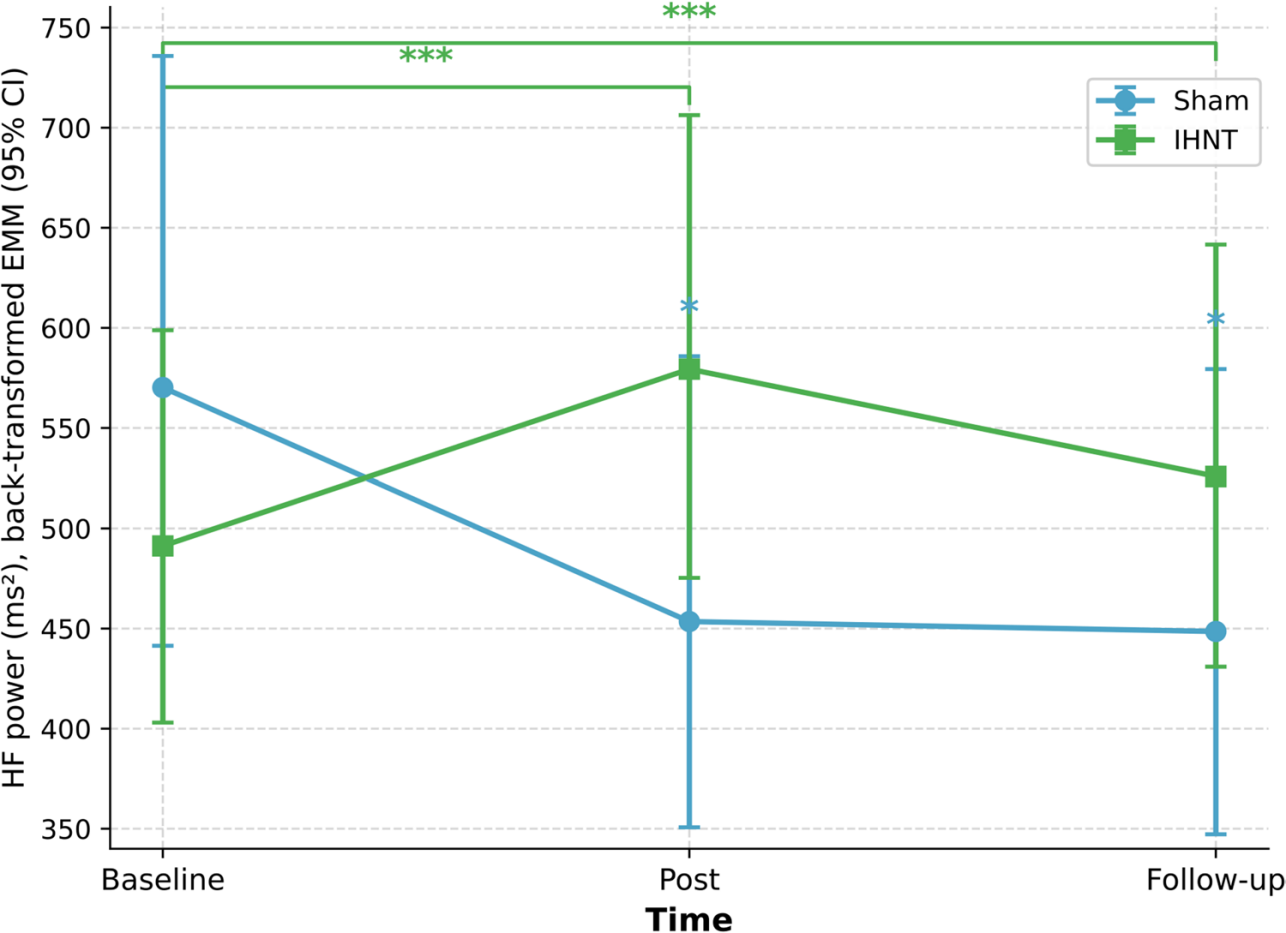
HF power profile by condition × time. Model-estimated marginal means (EMMs) with 95% confidence intervals for HF power, back-transformed from lnHF, at Baseline, Post-intervention, and Follow-up under IHNT and Sham. Estimates are from the prespecified REML linear mixed-effects model with an AR(1) residual structure and condition-specific heterogeneous residual variances. Symbols denote statistically significant within-condition changes versus Baseline based on nominal p values. No between-condition contrasts reached statistical significance after Holm–Bonferroni adjustment. *p < 0.05; ***p < 0.001

Within IHNT, lnHF increased from Baseline to Post-intervention (Δ = 0.165 ln units; +17.9%; back-transformed 95% CI + 16.0% to + 19.9%; *p* < 0.001) and remained elevated at Follow-up (+ 7.0%; back-transformed 95% CI + 5.0% to + 9.1%; *p* < 0.001). The Sham condition exhibited significant within-condition decreases at both Post-intervention (− 20.5%; back-transformed 95% CI − 35.6% to − 1.8%; *p* = 0.030) and Follow-up (− 21.3%; back-transformed 95% CI − 36.2% to − 2.9%; *p* = 0.030). Back-transformed estimated marginal mean profiles for HF power are shown in Fig. 4, with symbols indicating statistically significant within-condition changes versus Baseline. Between-condition contrasts did not reach statistical significance after Holm–Bonferroni adjustment: Baseline (− 13.8%; back-transformed 95% CI − 37.3% to + 18.4%; *p* = 0.355), Post-intervention (+ 27.8%; back-transformed 95% CI − 7.0% to + 75.8%; *p* = 0.130), and Follow-up (+ 17.2%; back-transformed 95% CI − 14.8% to + 61.3%; *p* = 0.325).

### Exploratory outcome: SDNN

For the exploratory global HRV index SDNN, a significant Condition × Time interaction was observed (χ²(2) = 7.19, *p* = 0.027; Supplementary Table S1A). Within IHNT, SDNN increased from 43.6 ms (EMM; SE = 3.4) to 46.5 ms (EMM; SE = 3.4) at Post-intervention (+ 2.96 ms [95% CI + 2.49 to + 3.43]; *p* < 0.001). No significant changes were detected in the Sham condition, and between-condition contrasts were non-significant at all time points (all *p* > 0.50).

### Sensitivity analysis: baseline-adjusted lnHF

The baseline-adjusted model (restricted to Post-intervention and Follow-up) confirmed a significant Condition × Time interaction (χ²(1) = 12.04, *p* < 0.001; Supplementary Table S3), with baseline lnHF as a strong predictor (χ²(1) = 324.13, *p* < 0.001). Adjusted estimated marginal means indicated numerically higher lnHF values for IHNT than Sham at Post-intervention (6.31 vs. 6.16 ln units; back-transformed: 549.5 vs. 475.1 ms²) and Follow-up (6.21 vs. 6.15; 499.2 vs. 468.5 ms²). However, IHNT-versus-Sham contrasts at individual time points were not statistically significant (Post: +15.6%; 95% CI − 10.2% to + 48.9%; *p* = 0.255; Follow-up: +6.5%; 95% CI − 17.4% to + 37.5%; *p* = 0.621).

### Individual response consistency

Directional consistency was assessed by inspecting individual change scores (ΔPost–Baseline) within each condition. Subject-level distributions and condition-specific variance patterns are reported in Supplementary Figure S1 and Supplementary Table S1.

### Training load and safety

Weekly cycling distance and training time were comparable between crossover periods (Supplementary Table S2). No sessions met prespecified termination criteria and no adverse events occurred throughout the study.

### Blinding efficacy

Overall guess accuracy was 52.4% (289/552 sessions), not different from chance (50%; binomial *p* = 0.287; mean confidence: 5.03 ± 1.63/10). Accuracy was near chance when stratified by condition (IHNT: 50.4%; Sham: 54.3%) and by period (Period 1: 50.0%; Period 2: 54.7%; all *p* > 0.13).

## Discussion

The principal finding of this randomized, sham-controlled 2 × 2 crossover trial is that IHNT at rest was associated with divergent cardiac autonomic trajectories compared with sham in master cyclists. Significant Condition × Time interactions were observed for lnRMSSD and lnHF, reflecting an upward trajectory in parasympathetic indices under IHNT and a concurrent downward trajectory under Sham. Taken together, these divergent patterns constitute a physiological signal consistent with a cardiac autonomic response to repeated normobaric hypoxic exposure. However, because pairwise between-condition contrasts did not remain significant after Holm–Bonferroni adjustment, this interpretation should be viewed as proof-of-concept rather than definitive evidence of point-specific superiority. These findings are consistent with the physiological rationale outlined in prior reviews of intermittent hypoxia as a cardiovascular intervention (Serebrovskaya and Xi 2016; Glazachev et al. 2021) and with original evidence of post-exposure vagal supercompensation in younger adults (Lee et al. 2025). They also provide preliminary evidence in endurance-trained master cyclists, a demographic in which age-related autonomic decline is well documented (Almeida-Santos et al. 2016; Lundstrom et al. 2023), but in which non-pharmacological hypoxic interventions targeting autonomic outcomes remain scarce (Timon et al. 2023).

### Parasympathetic reactivation and hormetic autonomic response

The within-IHNT increases in lnRMSSD (+ 13.6%) and lnHF (+ 17.9%) at Post-intervention are consistent with changes of potential physiological relevance, particularly given that IHNT involved no exercise component and participants were already aerobically trained. These magnitudes are consistent with the concept of hormetic stress, whereby repeated low-dose environmental challenges upregulate protective physiological responses (Mattson and Leak 2024). Specifically, intermittent hypoxic exposure activates peripheral chemoreceptors (Bernardi et al. 1998; Giles et al. 2016), and the subsequent normoxic recovery phase may favor autonomic recovery, potentially through mechanisms involving vagal re-engagement (Bernardi et al. 1998; Lee et al. 2025). Lee et al. (2025) recently reported post-exposure vagal supercompensation after a single session of low-dose normobaric hypoxia (FiO₂ = 13.5%) in healthy young adults, reflected by increases in RMSSD and HF power. Although direct quantitative comparison is limited by differences in population, exposure design, and analytical scaling, our findings are directionally consistent with this acute response and suggest that a similar autonomic pattern may emerge after repeated structured exposure over four weeks, with partial persistence after the intervention period.

The parallel behavior of lnRMSSD and lnHF supports the interpretation of a predominantly vagally mediated pattern (Task Force of the European Society of Cardiology and the North American Society of Pacing and Electrophysiology 1996; Shaffer and Ginsberg 2017). While HF power is strongly influenced by respiratory dynamics and should not be interpreted as a pure parasympathetic index, respiratory frequency and respiratory-centre activity can alter, and potentially increase, HF-band heart-period variability independently of baroreflex-mediated vagal modulation (Angelone and Coulter 1964; Saul et al. 1991; Porta et al. 2012). In this context, the concordance between lnHF and lnRMSSD, the latter being less susceptible to breathing-pattern confounding, strengthens the interpretation of a predominantly vagally mediated pattern but does not eliminate respiratory confounding.

A notable feature was the contrasting trajectory of the Sham condition, which exhibited significant within-condition decreases in lnHF (− 20.5% at Post, − 21.3% at Follow-up; both *p* = 0.030). This vagal withdrawal under sham is unexpected in a normoxic control group and may reflect cumulative fatigue (Plews et al. 2013; Buchheit 2014; Crowcroft et al. 2015) from the concurrent training program, seasonal effects, or subtle psychological disengagement from a perceived inactive intervention. These explanations remain speculative, as neither training intensity distribution, psychological expectancy, nor environmental co-factors were directly measured. Regardless of its origin, this bidirectional pattern amplifies the Condition × Time interaction and underscores the potential value of active sham controls in autonomic research.

### Between-condition contrasts and statistical power

The significant omnibus Condition × Time interactions (lnRMSSD: χ²(2) = 16.27, *p* < 0.001; lnHF: χ²(2) = 18.18, *p* < 0.001) constitute the primary inferential evidence in this trial, as they capture the overall divergence in temporal trajectories between conditions across all time points simultaneously. Pairwise between-condition contrasts at individual time points—a secondary, more conservative test—did not reach significance after Holm–Bonferroni adjustment (lnRMSSD: +13.4% at Post, *p* = 0.236; lnHF: +27.8% at Post, *p* = 0.130). This pattern is an expected statistical consequence of two compounding factors rather than an inferential inconsistency. First, the extreme residual heteroscedasticity observed across conditions (Sham/IHNT variance ratios: 84.94 for lnRMSSD, 164.84 for lnHF) substantially inflated the standard errors of between-condition contrasts. Second, in this analytical context, pairwise between-condition comparisons are estimated with lower precision because they combine within- and between-subject sources of variability (Jones and Kenward 2003). The non-significance of marginal contrasts therefore does not contradict the omnibus interaction; it reflects the reduced precision of point-specific estimates under conditions of marked variance asymmetry. Importantly, the study was primarily designed to assess the Condition × Time interaction within a crossover LMM framework, in which within-subject modelling improves statistical efficiency relative to parallel-group designs by accounting for between-participant variability. The point-specific between-condition contrasts, by contrast, were not the primary estimand and were more sensitive to the marked condition-specific residual variance asymmetry observed in this dataset.

The divergent-trajectories interpretation is further supported by the baseline-adjusted sensitivity analysis for lnHF, which confirmed the Condition × Time interaction (χ²(1) = 12.04, *p* < 0.001) after accounting for period-specific baseline values, making regression to the mean an unlikely primary driver of the observed pattern.

### Condition-specific variance patterns

A striking feature across all models was the markedly larger residual variance under Sham than under IHNT, with Sham/IHNT variance ratios of 84.94 for lnRMSSD and 164.84 for lnHF. These variance patterns primarily justify the adopted residual specification and should not be interpreted mechanistically in isolation. This disparity motivated the modelling of heterogeneous condition-specific residual variances and has two plausible interpretations. The hypoxic stimulus may engage relatively stereotyped chemoreflex pathways during recovery, producing more consistent day-to-day autonomic responses (Macoun et al. 2017; Krejčí et al. 2018). Alternatively, the variance difference may reflect greater susceptibility of the Sham condition to uncontrolled external influences (e.g., training variability, psychosocial stress). The present data cannot distinguish between these mechanisms; future work incorporating concurrent baroreflex sensitivity measures and objective training load metrics would clarify whether the dispersion pattern reflects genuine biological stabilization or methodological artifact.

### Practical implications for master athletes

From an applied perspective, the observed divergent autonomic trajectories may have relevance for training load management in aging endurance athletes, in whom autonomic decline reduces recovery capacity (Almeida-Santos et al. 2016; Hottenrott et al. 2019; Lundstrom et al. 2023). The within-IHNT improvements were partially sustained at Follow-up (lnRMSSD: +4.5%; lnHF: +7.0%), suggesting a transient autonomic pattern whose practical relevance for training management remains to be established. Furthermore, IHNT provides an autonomic stimulus without mechanical loading—a potentially valuable attribute when musculoskeletal stress is undesirable or contraindicated.

However, the present study did not assess performance outcomes, subjective recovery markers, or longer-term clinical endpoints. The translation of autonomic changes into functionally meaningful improvements remains speculative and requires longitudinal investigation. Any applied recommendations should therefore remain limited to the domain of autonomic modulation.

### Limitations

Several methodological strengths of this study—including the randomized crossover design, pre-registration, LMM with AR(1) and heterogeneous variances, and systematic blinding assessment—should be weighed against the following limitations.

First, the study was primarily designed to assess the Condition × Time interaction in a crossover LMM framework, the prespecified primary estimand, for which within-subject modelling provides gains in statistical efficiency over parallel-group designs by accounting for between-participant variability. The proof-of-concept sample (*n* = 23) was not, however, sized to achieve adequate precision for pairwise between-condition contrasts under the extreme residual heteroscedasticity observed (Sham/IHNT variance ratios: 84.94 for lnRMSSD, 164.84 for lnHF), which substantially inflated the standard errors of those secondary comparisons. Future trials should incorporate prespecified variance heterogeneity assumptions in their sample size calculations. Second, although FiO₂ was verified session-by-session (CV = 2.4%), the hypoxic generator and pulse oximeter lack independent validation against laboratory-grade instruments; however, consistent device use across conditions supports internal validity. Third, HRV outcomes capture basal autonomic state from standardized morning recordings rather than acute within-session responses, precluding inference about the dose-response trajectory across individual sessions. Fourth, participants self-managed their habitual training; despite comparable weekly volumes across periods (Supplementary Table S2), residual confounding by unmeasured fluctuations in training intensity cannot be excluded. Future studies should incorporate objective load metrics (e.g., power-based training stress score) and consider prespecified stratification by training load (Buchheit 2014; Sanders et al. 2017). Fifth, statistically significant period effects were observed (Perini et al. 1996) in the primary and secondary models (Tables 1 and 2), suggesting modest between-period shifts despite the washout; although accounted for in the crossover models, this may limit generalizability. Sixth, HRV was recorded during spontaneous breathing without formal respiratory control or respiratory frequency adjustment. Respiratory rate was neither monitored nor standardized across sessions, which precludes ruling out inter-session or inter-condition differences in breathing pattern as a contributing factor to the observed HF power changes. As noted above, respiratory influences on HF-band variability can operate independently of vagal modulation and should therefore be considered when interpreting the magnitude of lnHF changes.

Seventh, beyond respiratory rate per se, the HF power index used here is a univariate frequency-domain measure of heart-period variability and should not be interpreted as a direct measure of respiratory sinus arrhythmia or cardiorespiratory coupling. Bivariate approaches—such as transfer-function magnitude, cross-spectral coherence, or directional analyses between respiratory signals and RR intervals—provide complementary information by accounting for the respiratory input to heart-period oscillations (Saul et al. 1989; Abreu et al. 2023; Porta et al. 2024; Cairo et al. 2024). Because respiratory signals were not recorded in the present study, these analyses could not be performed and should be incorporated in future mechanistic trials.

Eighth, baroreflex sensitivity was not assessed. Therefore, the present design cannot determine whether changes in arterial pressure regulation or baroreflex-mediated vagal control contributed to the observed HRV trajectories. Future studies should incorporate concurrent blood pressure monitoring and baroreflex sensitivity assessment to clarify the contribution of baroreflex mechanisms to IHNT-related autonomic responses (Abreu et al. 2020).

## Conclusions

A 4-week IHNT intervention performed at rest was associated with divergent cardiac autonomic trajectories compared with sham in master cyclists, with consistent within-IHNT increases in lnRMSSD (+ 13.6%) and lnHF (+ 17.9%) at Post-intervention, partially sustained at Follow-up, alongside concurrent vagal withdrawal under Sham. These divergent patterns, supported by a significant Condition × Time interaction and a confirmatory baseline-adjusted sensitivity analysis, constitute a physiological signal consistent with a cardiac autonomic response to repeated normobaric hypoxic exposure. However, pairwise between-condition contrasts did not reach significance after Holm–Bonferroni adjustment, and the proof-of-concept nature of this trial precludes strong causal inference or applied recommendations beyond the domain of autonomic modulation. Confirmation in adequately powered trials incorporating performance and clinical endpoints is warranted.

## Supplementary Information

Below is the link to the electronic supplementary material.

Supplementary Material 1

Supplementary Material 2

Supplementary Material 3

Supplementary Material 4

## Data Availability

The deidentified analysis dataset (SPSS.sav) is available from the corresponding author upon reasonable request. The pre-registration, analytic code (R and SPSS), and supplementary materials are publicly available on the Open Science Framework (OSF; osf.io/2cgxj). Raw HRV time-series data cannot be shared publicly due to participant privacy agreements.
