# Supplementary material for: Intermittent normobaric hypoxia at rest is associated with divergent cardiac autonomic trajectories in master cyclists
Source: Eur J Appl Physiol. 2026 May 29;126(8):4615–27. doi: 10.1007/s00421-026-06280-z (PMC13451388; doi:10.1007/s00421-026-06280-z)
Supplement: Supplementary file 1 [file 421_2026_6280_MOESM4_ESM.docx]

**SUPPLEMENTARY FIGURES**

**Supplementary Figure S1.** Individual change scores for lnRMSSD and lnHF. Subject-level within-condition changes (Post–Baseline and Follow-up–Baseline) are displayed to summarize directional consistency. Direction-of-change is descriptive and does not imply clinical relevance or responder classification.

**Supplementary Figure S2.** Residual diagnostics for the primary lnRMSSD linear mixed-effects model. (A) Q–Q plot of standardized residuals. (B) Standardized residuals versus fitted values.

**Supplementary Figure S3.** Residual diagnostics for the primary lnHF linear mixed-effects model. (A) Q–Q plot of standardized residuals. (B) Standardized residuals versus fitted values.

# SUPPLEMENTARY TABLES

**Supplementary Table S1. Direction-of-change summary for lnRMSSD and lnHF.**

| Outcome | Change | Condition | n | % with Δ>0 | Median Δ [IQR] (ln units) |
| --- | --- | --- | --- | --- | --- |
| lnRMSSD | ΔPost–Baseline | IHNT | 23 | 100.0 | 0.122 [0.103, 0.154] |
| lnRMSSD | ΔPost–Baseline | Sham | 23 | 47.8 | -0.007 [-0.105, 0.110] |
| lnRMSSD | ΔFollow-up–Baseline | IHNT | 23 | 100.0 | 0.050 [0.021, 0.062] |
| lnRMSSD | ΔFollow-up–Baseline | Sham | 23 | 65.2 | 0.037 [-0.095, 0.156] |
| lnHF | ΔPost–Baseline | IHNT | 23 | 100.0 | 0.157 [0.137, 0.184] |
| lnHF | ΔPost–Baseline | Sham | 23 | 30.4 | -0.049 [-0.717, 0.012] |
| lnHF | ΔFollow-up–Baseline | IHNT | 23 | 100.0 | 0.076 [0.058, 0.088] |
| lnHF | ΔFollow-up–Baseline | Sham | 23 | 30.4 | -0.038 [-0.692, 0.026] |

**Supplementary Table S1A. SDNN (exploratory) — model summary, EMMs, and contrasts.**

| Fixed effect | Estimate (β) | SE | df | t/F | p | 95% CI L | 95% CI U | Effect size |
| --- | --- | --- | --- | --- | --- | --- | --- | --- |
| Condition | — | — | 1 | χ²=0.082 | 0.775 | — | — | — |
| Time | — | — | 2 | χ²=0.172 | 0.917 | — | — | — |
| Condition×Time | — | — | 2 | χ²=7.192 | 0.027 | — | — | — |
| Period | — | — | 1 | χ²=3.840 | 0.050 | — | — | — |
| CarryOver | — | — | 1 | χ²=0.257 | 0.612 | — | — | — |
| Residual variance ratio (Sham/IHNT) | 111.38 | — | — | — | — | — | — | — |
| Notes |  |  |  |  |  |  |  |  |

| Condition | Time | EMM (SDNN) | SE | 95% CI L | 95% CI U | Within-condition %Δ vs Baseline |
| --- | --- | --- | --- | --- | --- | --- |
| Sham | Baseline | 47.140 | 4.230 | 38.368 | 55.912 | 0.0% (ref) |
| Sham | Post | 45.090 | 4.248 | 36.280 | 53.900 | -4.3% |
| Sham | Follow-up | 47.025 | 4.249 | 38.214 | 55.837 | -0.2% |
| IHNT | Baseline | 43.579 | 3.423 | 36.479 | 50.679 | 0.0% (ref) |
| IHNT | Post | 46.538 | 3.435 | 39.415 | 53.662 | +6.8% |
| IHNT | Follow-up | 44.457 | 3.446 | 37.309 | 51.604 | +2.0% |

| Contrast | Δ (ms) | SE | df | t | p raw | p holm | 95% CI L | 95% CI U |
| --- | --- | --- | --- | --- | --- | --- | --- | --- |
| IHNT–Sham @ Baseline | -3.561 | 5.609 | 108 | -0.635 |  | 0.527 | -14.679 | 7.556 |
| IHNT–Sham @ Post | 1.448 | 5.644 | 108 | 0.257 |  | 0.798 | -9.738 | 12.635 |
| IHNT–Sham @ Follow-up | -2.569 | 5.665 | 108 | -0.453 |  | 0.651 | -13.798 | 8.660 |
| Sham: Post–Baseline | -2.050 | 2.198 | 108 | -0.933 |  | 0.706 | -7.047 | 2.947 |
| Sham: Follow-up–Baseline | -0.115 | 2.811 | 108 | -0.041 |  | 0.967 | -6.504 | 6.274 |
| IHNT: Post–Baseline | 2.960 | 0.208 | 108 | 14.208 |  | <0.001 | 2.486 | 3.433 |
| IHNT: Follow-up–Baseline | 0.878 | 0.266 | 108 | 3.296 |  | 0.001 | 0.272 | 1.483 |
| Condition×Time (Type III) | — | — | 2 | χ²=7.192 | 0.027 |  | — | — |
| Carry-over (Type III) | — | — | 1 | χ²=0.257 | 0.612 |  | — | — |

**Supplementary Table S2. Weekly external training load across crossover periods.**

| Variable | Period 1  Mean ± SD | Period 2  Mean ± SD | Paired difference (P2 − P1) | 95% CI (P2 − P1) | p (paired t) | Hedges’ g (paired) |
| --- | --- | --- | --- | --- | --- | --- |
| Weekly cycling distance (km·week⁻¹) | 325.18 ± 32.65 | 323.19 ± 31.52 | -1.99 | -9.78 to 5.80 | 0.601 | 0.109 |
| Weekly training time (h·week⁻¹) | 10.95 ± 1.27 | 10.95 ± 1.19 | -0.007 | -0.394 to 0.379 | 0.968 | 0.008 |

**Supplementary Table S3. Sensitivity (lnHF) — Baseline-adjusted model (Type III tests).**

| Effect | Chisq | df | p |
| --- | --- | --- | --- |
| (Intercept) | 84.90 | 1 | <0.001 |
| Condition | 0.68 | 1 | 0.409 |
| Time | 22.04 | 1 | <0.001 |
| Period | 6.10 | 1 | 0.013 |
| Carry-over | 2.55 | 1 | 0.110 |
| Baseline_lnHF | 324.13 | 1 | <0.001 |
| Condition:Time | 12.04 | 1 | <0.001 |

**Supplementary Table S4. Sensitivity (lnHF) — Baseline-adjusted EMMs.**

| Condition | Time | EMM | SE | df | LCL | UCL | ExpEMM | ExpLCL | ExpUCL |
| --- | --- | --- | --- | --- | --- | --- | --- | --- | --- |
| Sham | Post | 6.164 | 0.103 | 22 | 5.950 | 6.377 | 475.1 | 383.9 | 588.1 |
| IHNT | Post | 6.309 | 0.062 | 22 | 6.181 | 6.437 | 549.5 | 483.5 | 624.4 |
| Sham | Follow-up | 6.150 | 0.103 | 22 | 5.936 | 6.364 | 468.5 | 378.3 | 580.3 |
| IHNT | Follow-up | 6.213 | 0.062 | 22 | 6.084 | 6.342 | 499.2 | 438.8 | 567.9 |

**Supplementary Table S5. Sensitivity (lnHF) — Baseline-adjusted between-condition contrasts.**

| Time | contrast | Estimate | SE | df | LCL | UCL | t | p | PctDelta | PctLCL | PctUCL |
| --- | --- | --- | --- | --- | --- | --- | --- | --- | --- | --- | --- |
| Post | IHNT - Sham | 0.145 | 0.127 | 63 | -0.107 | 0.398 | 1.15 | 0.255 | 15.6 | -10.2 | 48.9 |
| Follow-up | IHNT - Sham | 0.063 | 0.128 | 63 | -0.192 | 0.318 | 0.50 | 0.621 | 6.5 | -17.4 | 37.5 |

**Supplementary Table S6. Sensitivity analysis for lnRMSSD excluding two outlying observations.**

| Effect | Full model: F (df1, df2) | Full model: p | Outlier-excluded: F (df1, df2) | Outlier-excluded: p |
| --- | --- | --- | --- | --- |
| Condition | 0.027 (1, 35.125) | 0.871 | 0.024 (1, 41.037) | 0.877 |
| Time | 3.691 (2, 105.482) | 0.028 | 0.832 (2, 98.549) | 0.438 |
| CarryOver | 2.227 (1, 25.068) | 0.148 | 1.177 (1, 26.183) | 0.288 |
| Condition × Time | 8.671 (2, 99.731) | <0.001 | 7.334 (2, 98.721) | 0.001 |

**Supplementary Table S7. lnRMSSD — Planned contrasts (Δln) and Holm-adjusted p-values.**

| **Contrast** | **Δ (ln)** | **SE** | **df** | **t** | **p value** | **95% CI L** | **95% CI U** | **Back-transform (%Δ)** |
| --- | --- | --- | --- | --- | --- | --- | --- | --- |
| IHNT–Sham @ Baseline | -0.071 | 0.105 | 108 | -0.681 | 0.497 | -0.279 | 0.136 | -6.9% [-24.3, +14.6] |
| IHNT–Sham @ Post | 0.125 | 0.105 | 108 | 1.191 | 0.236 | -0.083 | 0.334 | +13.4% [-8.0, +39.7] |
| IHNT–Sham @ Follow-up | 0.003 | 0.106 | 108 | 0.024 | 0.981 | -0.207 | 0.212 | +0.3% [-18.7, +23.6] |
| Sham: Post–Baseline | -0.069 | 0.051 | 108 | -1.353 | 0.358 | -0.186 | 0.047 | -6.7% [-17.0, +4.8] |
| Sham: Follow-up–Baseline | -0.030 | 0.061 | 108 | -0.489 | 0.626 | -0.169 | 0.109 | -3.0% [-15.6, +11.6] |
| IHNT: Post–Baseline | 0.127 | 0.0056 | 108 | 22.820 | <0.001 | 0.115 | 0.140 | +13.6% [+12.1, +15.0] |
| IHNT: Follow-up–Baseline | 0.044 | 0.0067 | 108 | 6.600 | <0.001 | 0.029 | 0.059 | +4.5% [+2.9, +6.1] |

Notes. Holm adjustment applies to between-condition contrasts; within-condition contrasts are nominal. Back-transformation: %Δ = exp(Δln) − 1.

**Supplementary Table S8. lnHF — Planned contrasts (Δln) and Holm-adjusted p-values.**

| **Contrast** | **Δ (ln)** | **SE** | **df** | **t** | **p value** | **95% CI L** | **95% CI U** | **Back-transform (%Δ)** |
| --- | --- | --- | --- | --- | --- | --- | --- | --- |
| IHNT–Sham @ Baseline | -0.149 | 0.160 | 108 | -0.928 | 0.355 | -0.466 | 0.169 | -13.8% [-37.3, +18.4] |
| IHNT–Sham @ Post | 0.245 | 0.161 | 108 | 1.528 | 0.130 | -0.073 | 0.564 | +27.8% [-7.0, +75.8] |
| IHNT–Sham @ Follow-up | 0.159 | 0.161 | 108 | 0.988 | 0.325 | -0.160 | 0.478 | +17.2% [-14.8, +61.3] |
| Sham: Post–Baseline | -0.229 | 0.093 | 108 | -2.473 | 0.030 | -0.440 | -0.019 | -20.5% [-35.6, -1.8] |
| Sham: Follow-up–Baseline | -0.240 | 0.106 | 108 | -2.264 | 0.030 | -0.480 | 0.001 | -21.3% [-38.1, +0.1] |
| IHNT: Post–Baseline | 0.165 | 0.0072 | 108 | 22.875 | <0.001 | 0.149 | 0.181 | +17.9% [+16.0, +19.9] |
| IHNT: Follow-up–Baseline | 0.068 | 0.0083 | 108 | 8.242 | <0.001 | 0.049 | 0.087 | +7.0% [+5.0, +9.1] |

Notes. Holm adjustment applies to between-condition contrasts; within-condition contrasts are nominal. Back-transformation: %Δ = exp(Δln) − 1.

Supplementary Table S9. lnRMSSD — Estimated marginal means (EMMs) by Condition × Time.

| **Condition** | **Time** | **EMM (ln)** | **SE** | **95% CI L** | **95% CI U** | **%Δ vs BL** |
| --- | --- | --- | --- | --- | --- | --- |
| Sham | Baseline | 3.927 | 0.079 | 3.763 | 4.092 | 0.0% (ref) |
| Sham | Post | 3.858 | 0.080 | 3.692 | 4.023 | -6.7% |
| Sham | Follow-up | 3.897 | 0.080 | 3.732 | 4.063 | -3.0% |
| IHNT | Baseline | 3.856 | 0.064 | 3.723 | 3.989 | 0.0% (ref) |
| IHNT | Post | 3.983 | 0.064 | 3.850 | 4.117 | +13.6% |
| IHNT | Follow-up | 3.900 | 0.064 | 3.766 | 4.034 | +4.5% |

Notes. EMMs are on the ln scale; percentage changes are back-transformed within-condition changes relative to the corresponding Baseline (%Δ = exp(Δ) − 1).

Supplementary Table S10. lnHF — Estimated marginal means (EMMs) by Condition × Time.

| **Condition** | **Time** | **EMM (ln)** | **SE** | **95% CI L** | **95% CI U** | **%Δ vs BL** |
| --- | --- | --- | --- | --- | --- | --- |
| Sham | Baseline | 6.346 | 0.123 | 6.090 | 6.601 | 0.0% (ref) |
| Sham | Post | 6.117 | 0.124 | 5.860 | 6.373 | -20.5% |
| Sham | Follow-up | 6.106 | 0.123 | 5.850 | 6.362 | -21.3% |
| IHNT | Baseline | 6.197 | 0.095 | 5.999 | 6.395 | 0.0% (ref) |
| IHNT | Post | 6.362 | 0.096 | 6.164 | 6.560 | +17.9% |
| IHNT | Follow-up | 6.265 | 0.096 | 6.066 | 6.464 | +7.0% |

Notes. EMMs are on the ln scale; percentage changes are back-transformed within-condition changes relative to the corresponding Baseline (%Δ = exp(Δ) − 1).
